# An insight into pathogenicity and virulence gene content of *Xanthomonas* spp. and its biocontrol strategies

**DOI:** 10.1016/j.heliyon.2024.e34275

**Published:** 2024-07-08

**Authors:** Riddha Dey, Richa Raghuwanshi

**Affiliations:** Department of Botany, Mahila Mahavidyalaya, Banaras Hindu University, Varanasi, 221005, Uttar Pradesh, India

**Keywords:** *Xanthomonas*, Pathogenicity, Biocontrol, Virulence genes

## Abstract

The genus *Xanthomonas* primarily serves as a plant pathogen, targeting a diverse range of economically significant crops on a global scale. *Xanthomonas* spp. utilizes a collection of toxins, adhesins, and protein effectors as part of their toolkit to thrive in their surroundings, and establish themselves within plant hosts. The bacterial secretion systems (Type 1 to Type 6) assist in delivering the effector proteins to their intended destinations. These secretion systems are specialized multi-protein complexes responsible for transporting proteins into the extracellular milieu or directly into host cells. The potent virulence and systematic infection system result in rapid dissemination of the bacteria, posing significant challenges in management due to complexities and substantial loss incurred. Consequently, there has been a notable increase in the utilization of chemical pesticides, leading to bioaccumulation and raising concerns about adverse health effects. Biological control mechanisms through beneficial microorganism (*Bacillus*, *Pseudomonas*, *Trichoderma*, *Burkholderia*, AMF, etc.) have proven to be an appropriate alternative in integrative pest management system. This review details the pathogenicity and virulence factors of *Xanthomonas*, as well as its control strategies. It also encourages the use of biological control agents, which promotes sustainable and environmentally friendly agricultural practices.

## Glossary

**AMF**: Arbuscular mycorrhizal fungi
**BCA**: Biological control agents
**EPS**: Exopolysaccharides
**ETI**: Effector-Triggered Immunity
**Hrp:** Hypersensitive response and pathogenicity
**LPS**: Lipopolysaccharides
**M/DAMP-TI**: Microbe/Damage Associated Molecular Pattern-Triggered Immunity
**MDCA**: 3,4-(methylenedioxy) cinnamic acid
**NRPS-PKS**: Non-Ribosomal Peptide Synthetase-Polyketide Synthase
**PTI**: Pathogen-Triggered Immunity
**RTX**: Repeats-in-toxins
**T1SS**: Type I Secretion System
**T2SS**: Type II Secretion System
**T3E**: T3 effector
**T3SS**: Type III Secretion System
**T4SS**: Type IV Secretion System
**T5SS**: Type V Secretion System
**T6SS**: Type VI Secretion System
**TALE**: Transcription activator-like effectors
**Xop:** *Xanthomonas* outer proteins

## 1 Introduction

*Xanthomonas* is a Gram-negative, yellow-colored, plant-associated bacterium and is classified under the Gammaproteobacteria, comprising about 30 species that cause severe diseases in around 400 plant hosts. This includes economically important crops, like rice, tomato, citrus, pepper etc. This phytopathogen shows high specificity towards the host plant and tissue. It invades xylem elements and intercellular space between mesophyll parenchyma tissue. *Xanthomonas* genomes encompass different Mobile Genetic Elements (MGEs), which are major factors for virulence, genetic variations, and genome structure [1]. Examples of MGEs are transposons (Tn), Genomic Islands (GI), Insertion Sequence (IS), and plasmids. Genomic islands in *Xanthomonas* spp. serve multifaceted functions that are intertwined with bacterial pathogenicity, persistence, and evolutionary processes [1]. Recombination and horizontal gene transfer play pivotal roles in shaping the population structure and fostering diversity within the pathosystems of *Xanthomonas* [2]. *Xanthomonas* possess six main secretion systems among which the type III secretion system plays a substantial role in suppressing host defenses and promoting the progression of the disease. A multitude of factors like type III effectors, adhesins, lipopolysaccharides, and transcription factors, are identified to be critical elements regarding host specificity and bacterial pathogenicity [3]. Comprehending the pathogenicity of *Xanthomonas* has consistently posed a challenge because of its extensive host range and intricate infection pathways. Functional and comparative genomic studies have proven invaluable in uncovering how this bacterial group has evolved to exploit an exceptionally wide array of plant hosts. Enhanced insights into the pathogenic adaptations exhibited by *Xanthomonas* spp. and their infection pathways hold the potential to drive advancements in the prevention and management of bacterial diseases of plants.

This review focuses on an elaborate study of the lifecycle of *Xanthomonas* spp. and consolidates the latest developments encompassing the macromolecular secretion systems, ranging from type I to type VI (T1SS to T6SS), as they pertain to *Xanthomonas* spp. This also includes an overview of chemical and biological control methods for the agricultural management of *Xanthomonas* to mitigate the occurrence of diseases.

### 1.1 *Xanthomonas* lifestyle

The genus *Xanthomonas* is known for its phytopathogenic diversity. It belongs to the family Xanthomonadaceae, subclass Gammaproteobacteria, and is a Gram-negative bacterial genus. It also has a contrasting phenotypic uniformity. The *Xanthomonas* colonies are morphologically yellow due to membrane-bound xanthomonadin pigment, which protects them from photobiological damage [[4], [5], [6]]. Initially, members of the *Xanthomonas* genus were classified into distinct species based on their infection capability to specific hosts. However, following classical naming conventions, the majority of these species were subsequently consolidated into a singular entity called *Xanthomonas campestris* (*X*. *campestris*) [7]. This collective group was further categorized into various pathovars. Nonetheless, owing to multiple taxonomic revisions, there remains ongoing debate regarding the nomenclature of the roughly 19 species and 4140 pathovars within the *Xanthomonas* genus [8].

Different *Xanthomonas* strains cause bacterial disease in more than 400 hosts, including cabbage, tomato, cassava, wheat, rice, pepper, citrus, and beans [9]. Although they show varied disease phenotypes and diverse host ranges, the maximum similarity is observed at the genomic level. Most of the genes are conserved sequences. A single phylogenetic group was represented by the first two sequenced *X. perforans* strains discovered in Florida in the 1990s [2,10]. This genus has been diversely studied due to its economic loss and large genetic diversity. *X. citri* subsp. *citri* causal organism for citrus canker, which is broadly spread around the world, especially in Brazil, USA, Mexico, India, and Japan [11]. *X. campestris* pv. *campestris* is the most destructive phytopathogen responsible for causing black rot disease. The infection results in the darkening of vascular tissue and the development of foliar lesions, which can vary from yellowing to tissue death (necrosis). This leads to vegetable decay and renders it unsuitable for consumption [12].

It causes significant economic losses to the Brassicaceae family including cabbage, broccoli, cauliflower, and kale [13]. Bacterial leaf spot is a highly challenging disease observed in tomato and pepper resulting in a great economic loss. The causal agents include *X. vesicatoria*, *X. euvesicatoria*, *X. perforans*, and *X. gardneri* [14,15]. *Xanthomonas* wilt, caused by *X. campestris* pv. *musacearum*, is a significant disease that has spread globally. It was initially observed in Ensete in 1968 [16] and later in bananas in Ethiopia in 1974 [17]. Since 2001, it has been frequently reported in various countries, including Kenya [18], Tanzania [19], Uganda [20], the Democratic Republic of the Congo [21], Rwanda, Burundi [18]. The disease has lately been recorded in the Democratic Republic of the Congo in the South Kivu communities of Uvira and Fizi, in the northern Katanga territory of Kalemie, and in the Tshopo area of Oriental Province.

**Fig. 1:**
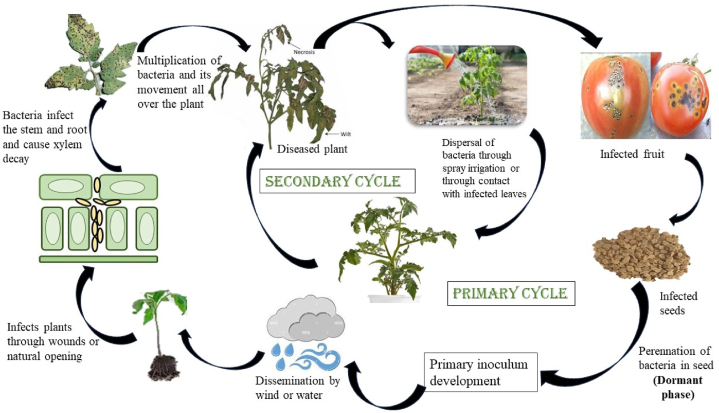
Life cycle of *Xanthomonas* spp.

*Xanthomonas* exhibit both epiphytic and endophytic life cycles, which involve distinct interactions with their host plants as depicted in Fig. 1. The epiphytic life cycle of *Xanthomonas* refers to the phase of the bacteria's lifecycle when it resides on the external surfaces of plant tissues without causing an active infection. It starts with adhesion and colonization. They proceed to infiltrate the host tissue through natural openings or injuries on the plant's surface. Conventionally, they are exposed to harsh environmental conditions on the surface of plants [22]. Despite these challenges, *Xanthomonas* is capable of persisting on leaf surfaces for prolonged durations [23]. The survival time of *Xanthomonas* on leaf surfaces is not certain; it can vary from a few weeks to several months [23]. Kayaaslan et al. [24], stated that *Xanthomonas* spp. have the ability to persist epiphytically on seeds, and it can be viable for up to 18 months. Once attached to the surface, the bacteria start to aggregate and form microcolonies on the plant's surface. These microcolonies provide protection from environmental stresses, such as desiccation and UV radiation. These microcolonies can develop into more complex structures known as biofilms to provide additional protection and help bacteria withstand harsh conditions [25]. *Xanthomonas* biofilm is a dynamic structure, and both its formation and dispersal are regulated by the quorum sensing signal molecule known as the diffusible signal factor (DSF). DSF, in conjunction with the internal secondary messenger cyclic di-GMP, plays a role in promoting biofilm formation by inducing the production of exopolysaccharides (EPS) and the assembly of pili [26].

The primary constituent of the biofilm matrix in *Xanthomonas* spp. is typically Xanthan gum [27]. During the endophytic life cycle, they gain access to the host tissue through natural openings (hydathodes, stomata, and wounds). Bacterial cells need to adjust their physiology according to the particular conditions they confront during colonization, whether it is within the host mesophyll (for non-vascular pathogens) or xylem vessel tissues (for vascular pathogens). Subsequently, they progress by migrating towards the central veins for multiplication during which bacteria proliferate within the host tissue. As the bacterial population increases, they resurface on the leaf's exterior. Through dispersion via rain and wind, they find their way into a new host, initiating a fresh infection cycle. When the bacterial population reaches a critical threshold, they invade the mesophyll tissues, giving rise to the characteristic disease symptoms on the leaves [28].

### 1.2 Virulence factors

**Fig. 2:**
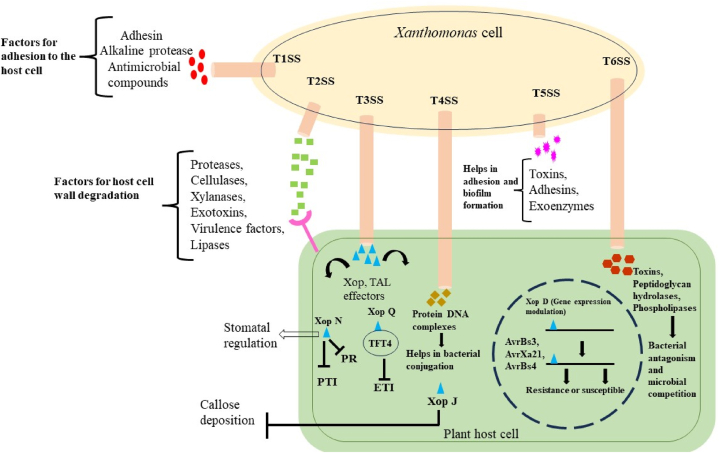
Mechanism of action of secretion systems of *Xanthomonas* spp.

The severity of pathogen infection is virulence. The virulence and pathogenicity imparting genes and gene clusters in *Xanthomonas* spp. consist of six types of protein secretion systems. Each protein type differs in function and composition [29] and plays an important role in host-pathogen interaction (Fig. 2).

### 1.3 Type I secretion system (T1SS)

The main role of the T1SS is to secrete unfolded cognate substrates directly from the cytoplasm to the extracellular medium involving an intermediary periplasmic state [30] which results in adhesion to the host cell. The T1SS is composed of a heterotrimeric protein complex, which includes an ATP-binding cassette transporter in the inner leaflet, a protein channel in the outer leaflet, and a membrane fusion protein that bridges the inner and outer leaflet. *RaxST* (tyrosine sulfotransferase), *RaxA* (membrane fusion protein), *RaxB* (adenosine triphosphate–binding cassette transporter), *RaxC* (outer membrane protein) marked the existence of T1SS [31]. These proteins recognize effectors containing double glycine leader peptide. It was reported that in *X. perforans*, *raxST*, *raxA*, and *raxB* are encoded in a same operon [32] whereas the *raxC* is located outside the *raxSTAB* operon [33]. The *raxABC* T1SS is regulated by *raxH* and *raxR* which are located downstream of the *raxSTAB* operon [34]. It was stated that on mutating the *raxX* gene, the virulence in rice was impaired, concluding that it promotes infection of *X. oryzae* pv. *oryzae* [35]. T1SS substrates belong to three different groups including bacteriocin, proteins with non-peptide RTX motif (Repeats in Toxins), and large adhesin containing RTX at the C-terminal. RTX proteins make up the majority of T1SS effectors. It includes proteases, adhesins, lipases, toxins, and may feature different quantities of RTX motifs along with an uncleavable T1SS-specific C-terminal secretion signal. RTX proteins are conveyed in their unfolded state, and once they are secreted, extracellular Ca^2+^ binds to the RTX motif, prompting their folding [36]. Fonseca et al. [37], reported that in *X. citri* pv. *citri* 306 has *hlyDB* T1SS that secretes the two RTX-related effectors XAC2197 and XAC2198 which plays role in its pathogenicity. However, it was also observed that *X. citri* pv. *aurantifolii* genome lacks the *hlyDB* T1SS, which makes it less aggressive than *X. citri* pv. *citri*.

### 1.4 Type II secretion system (T2SS)

T2SS is crucial for secreting enzymes like proteases, lipases, cell wall lyase enzymes, and xylanases which results in host cell wall degradation. It also transports protein from *Xanthomonas* periplasm to the extracellular milieu. The T2SS apparatus comprises a total of 12 proteins, which are responsible for various functions. These proteins include a cytoplasmic ATPase known as *gspE*, an inner membrane platform complex composed of *gspC*, *gspF*, *gspL*, and *gspM*, an outer membrane channel represented by *gspD*, a prepilin peptidase called *gspO*, and a pseudopilus formed by *gspG*, *gspH*, *gspI*, *gspJ*, and *gspK*. Together, these components work in concert to eject the substrate. Recent transcriptome analysis indicates that the *gspD* mutant in the *X. translucens* pv. *cerealis* strain NXtc01 exhibits a marked reduction in disease development in wheat, highlighting the pivotal role of the T2SS in enhancing virulence [38]. The secretin protein family forms a complex transmembrane channel in the outer membrane. Substances are secreted through this channel via a periplasmic pilus, which undergoes constant assembly and disassembly, effectively propelling T2SS substrates [39].

In the *Xanthomonas* genus, there are two distinct gene clusters associated with T2SS: *xps*, which is found consistently across the entire genus, and *xcs*, which is present alongside *xps* in certain species like *X. citri* and *X. campestris* but is absent in others, including *X. oryzae* strains [40,41]. In *X. perforans*, the T2SS is encoded by both the *xps* and *xcs* gene clusters [42]. While xps in T2SS is widely considered the primary mechanism for secreting effectors and influencing virulence in various *Xanthomonas* species, the role of xcs T2SS remains unclear as it has not been associated with any specific function or role to date [43,44]. Nonetheless, a recent RNA-seq investigation carried out by Zhang et al., [45] showed that when the genes for stringent response regulators like the ppGpp synthases *SpoT* and *RelA* and transcription factor *DskA* are mutated in *X. citri* pv. *citri*, it led to a significant reduction in *xcs* gene expression, while *xps* genes remained unaffected. This finding raised curiosity because the stringent response typically played a role when the organism faces stressors like limited nutrients. It suggested a potential, albeit unexplored, role for this enigmatic T2SS in aiding cell survival during challenging environmental conditions. In *X. euvesicatoria*, the T2SS contributes to enhancing virulence, and its activity is governed by master regulators *HrpX* and *HrpG*, which also control the T3SS [46]. While the detailed role of T2SS in pathogenicity is not thoroughly elucidated in *X. perforans*, it is speculated to be comparable to its function in *X. euvesicatoria*.

### 1.5 Type III secretion system (T3SS)

T3SS plays the main role in pathogenesis. Effector protein translocation inside host cells and pilus structure assembly are the major virulence factors enabling host specialization and infection [47]. This system alters the behavior of the host cell and directly delivers the T3 effector (T3E) proteins into it through Hrp pilus. The core secretion apparatus is conserved among pathogenic bacteria. T3SS in *Xanthomonas* spp is encoded by the hypersensitive response and pathogenicity (hrp) cluster, which in turn is regulated by *hrpX* and *hrpG* master switches, consisting of an array of genes organized in transcriptional units [48]. The Hrp pilus spans the host plant cell wall and connects the T3SS translocon. Individual translocon components mutate, leading to reduction or loss of pathogenicity. The T3E protein translocation plays a vital role in both bacterial cell proliferation and the emergence of disease symptoms [[49], [50], [51]]. Colonization of the host milieu is promoted by T3E proteins, which suppress the immune response. The T3E *Xanthomonas* spp. is a member of the AvrBs3/PthA family. The absence of either pthA or AvrBs3 from their respective strains led to a significant decrease in the extent of infection of pathogen replication capacity within the host tissue leading to reduced disease symptoms. The existence of the *Xop* effector gene represents a distinct characteristic specific to *Xanthomonas* spp. Yan et al. [52] discovered the *Min* system, composed of *MinC*, *MinD*, and *MinE* proteins, constituted a regulatory mechanism that negatively influenced bacterial cell division. This system is encoded by the genes *minC*, *minD*, and *minE* and is involved in the negative regulation of *hrp*B1 and *hrp*F, in *X. oryzae* pv. *oryzae*. Advanced approaches like machine learning have been developed for detailed searching of T3E in *Xanthomonas* genomes based on parameters like GC content, codon usage, gene homology, structural patterns, amino-terminal secretion signals, and *HrpG/HrpX* signaling pathway [53]. Effectors play a dual role in *Xanthomonas* biology, being not only crucial for pathogenicity but also acting as factors that determine host specificity and contribute to pathogen fitness.

### 1.6 T3SS effectors

**TAL effectors:** Transcription activator-like effectors (TALE) are a discrete family of T3E proteins present in most of the species of the genus *Xanthomonas*, involved in enhancing the bacterial plasticity to host plants. The rearrangeable repetitive domains aid the binding of promoters of host susceptibility genes sequence-specifically [54,55]. The rearranged central-repeat domains of TAL proteins recognize EBE (Effector Binding Elements) situated in promoters [56]. Further, TALEs are not observed in *X. gardneri*, *X. campestris,* and *X. euvesicatoria* [10,57]. However, TALEs are predominant in *X. oryzae* [58]. Additionally, *X. translucens* pv. *undulosa* deploys the TALE Tal8 that modifies the abscisic acid pathway to increase disease susceptibility in wheat [59] and *X. oryzae* pv. *oryzae*. TALE targets the sugar transporters (encoded by SWEET genes) in rice leading to bacterial blight disease. *OsSWEET11*/*Os8N3*/*Xa13* was the first gene discovered among the *SWEET* gene family involved in host-bacterial pathogen interaction in bacterial blight of rice caused by *X. oryzae* pv. *oryzae* [60,61]. The rice plants with silenced *OsSWEET11* showed resistance towards *Xanthomonas oryzae* pv. *oryzae* [61]. *X. oryzae* pv. *oryzae* strain PXO99^A^ released a distinct type III TAL effector PthXo1, which attached to the promoter of *OsSWEET11* in rice, thereby triggering its transcription [62]. Streubel et al. [63], proposed that PthXo3 (TalBH), TalC, and AvrXa7 targeted *OsSWEET14*/*Os11N3*. This resulted in the accumulation of sugar in the apoplast and xylem that led to bacterial colonization [64,65]. In *X. oryzae* pv. *oryzae* TALE (PthXo1 and PthXo2) target *OsSWEET11* and *OsSWEET13* [65]. The *SWEET* gene (*GhSWEET10*) belonging to clade III in *Gossypium* sp. (cotton) similarly made the plant susceptible to *X*. *citri* subsp. *malvacearum* infection, which is targeted by the TAL effector Avrb6 [66]. TAL20_Xam66_, secreted by *X*. *axonopodis* pv. *manihoti*, the causal agent of bacterial blight, induced the expression of *MeSWEET10a* in *Manihot esculentum* (cassava), aiding the pathogen in obtaining hexose from the host plants [67]. *X*. *citri* subsp. *citri* secretes TAL as a component of its infection strategy, stimulating the expression of *CsSWEET1* in *Citrus aurantiifolia* (lime), thereby leading to the development of bacterial canker disease [68]. To date, only clade III *SWEET* genes have been identified as targets of TAL effectors produced by bacterial pathogens.

### 1.7 Xop (*Xanthomonas* outer proteins)

*Xanthomonas* is exposed to host immune responses after entering the plant tissues. Some examples of the responses are ETI (Effector-Triggered Immunity) and M/DAMP-TI (Microbe/Damage Associated Molecular Pattern-Triggered Immunity [69]. T3SS effectors (T3E) are Xop proteins or Avr (avirulence), that aid *Xanthomonas* to cope with the immune responses. These Xop are delivered inside the host cell using T3SS [69]. There are 53 Xop families (XopA- Xop BA) identified to date, which are elaborately discussed by White et al. [70]. Further, the PTI pathways are targeted by *Xanthomonas* T3Es such as XopR, XopK, XopP, XopN, XopL, XopS, XopB, and AvrAC, AvrBs2 [71,72] whereas DAMP-TI is inhibited by XopQ, XopX, XopZ, and XopN. ETI and PTI responses are interfered by XopH and XopQ [31,73]. XopD and XopJ disrupt the host's ubiquitin proteasomal system, leading to suppressing immunity targeting the PTI factors and defense mediated by salicylic acid [31]. XopD consists of SUMO protease (Small Ubiquitin-like Modifier) (C48 protease family member). XopD is responsible for the de-sumoylation process from SUMO-modified plant proteins [74,75]. It is also observed that XopD is nuclear localized and has nonspecific DNA binding activity [74,76]. Teper et al. [77] proposed that XopD possesses a helix-loop-helix domain essential for DNA binding, along with two conserved ERF-associated amphiphilic motifs that suppress transcription induced by salicylic acid and jasmonic acid in plants. Also, XopD inhibits the development of symptoms during the latter phases of infection in tomato leaves. Enhanced population and genomic databases as well as machine-learning techniques, have advanced the ability to identify and understand Xops and their interactions with hosts [78].

### 1.8 Type IV secretion system (T4SS)

The primary function of the T4SS is to facilitate the transfer of proteins and protein-DNA complexes into specific target cells. This system is responsible for various processes, including bacterial conjugation, delivery of T-DNA into *A*. *tumefaciens*, the transmission of protein-based effectors into host cells as well as the conveyance of harmful effectors into the cells of competing bacterial species. In *Xanthomonas* spp. two classes of T4SS are present, the established T4ASS and T4BSS [42]. T4BSS is larger and more complex than T4ASS. *Xanthomonas* spp. carries genes responsible for encoding the essential 12 subunits present in T4ASS. These subunits can be grouped into three main categories: subunits responsible for the outer membrane pore formation, VirB7, VirB9, and VirB10; subunits responsible for inner membrane pore formation, VirB3, VirB4, VirB6, VirB8, VirB11, VirD4, and the N-terminal domain of VirB10; and subunits VirB2 and VirB5, are anticipated to create an extracellular pilus structure. Additionally, VirB1 is predicted to function as a periplasmic lytic transglycosylase, contributing to peptidoglycan remodeling during the biogenesis of the T4SS [79]. Nonetheless, recent research in *X. citri* has revealed that a T4SS located on a plasmid can transfer toxic effectors into neighboring bacteria during competition, leading to the demise of those other bacterial cells [80]. VirD4, an ATPase located in the inner membrane, identifies and transports harmful substances through a shared C-terminal domain known as ‘*Xanthomonas* VirD4 interacting protein conserved domains’ (XVIPCDs), utilizing the T4SS pilus as a conduit [31]. The transport of phytotoxins ultimately disrupts the electric potential across the plant membrane, culminating in the demise of plant cells [81,82]. *X. oryzae* pv. *oryzae* strain PXO99^A^ lacks the entire T4SS.

### 1.9 Type V secretion system (T5SS)

The primary function of the T5SS is to transport effectors, including adhesins, enzymes, and toxins. It shows non-fimbrial adhesins. These effectors have a significant role in processes such as adhesion to both host and non-host plants, the formation of biofilms, and the aggregation of cells [31]. It is structurally simple consisting of two main domains: β-barrel present in the outer membrane and a secreted passenger protein. T5SS have been categorized into subclasses (Va to Ve), with a newly described Vf subclass identified specifically in *H. pylori*. This does not require sources of energy such as ATP or electrochemical gradients for transportation [83]. Va plays the role of auto-transporter, whereas Vb is known as TPS (Two Partner Secretion System) constituted of a translocator protein and a cognate passenger protein [84]. The transportation process to periplasm from the cytoplasm is carried out through Sec translocase pathway [85]. Vc are known as TAA (Trimeric Autotransporter Adhesins) and remain in the outer membrane. The exported effectors either get released to the extracellular environment or remain attached to the transporter. Current research strongly indicates that labeling T5SS as “autotransporters” is inaccurate. These proteins, in fact, necessitate the involvement of numerous external factors to facilitate their export to the bacterial cell surface, with distinct requirements at various stages of the process [86].

### 1.10 Type VI secretion system (T6SS)

The primary function of T6SS is to facilitate antagonistic behaviour by delivering toxic substances. This system is classified into five clades (i1 to i5) and is present in all bacterial species that are associated with plants, whether it's pathogenic or non-pathogenic. T6SS is much more involved in bacterial competition rather than virulence. It confers advantages in terms of fitness by enhancing competitiveness against other members of the bacterial community [87]. There are three main structures of T6SS in the cell envelope, which accumulate together to function in the contractile system: a membrane complex, a baseplate, and an inner tube extension enveloped by a contractile sheath. Recent studies proved that T6SS is subdivided into three main clusters on the basis of gene content; T6SS-I, T6SS-II, and T6SS-III. T6SS-I were found in *X. phaseoli, X. euvesicatoria, X. oryzae, X. axonopodis,* and *X. vasicola*. Only three species including *X. oryzae, X. hortorum*, and *X. fragarie* contain a full T6SS-II cluster. T6SS-III is present in *X. citri* pv. *citri* and phylogenetically related species. *X. maliensis* is an exception as it has complete T6SS-I and partial T6SS-II and T6SS-III clusters [88]. Moreover, according to studies by Choi et al. [89] and Zhu et al. [90], the removal of *hcp* in T6SS-2 had a partial detrimental effect on the virulence of *X. oryzae* pv. *oryzae* and *X. oryzea* pv. *oryzicola*. However, when *hcp* was deleted in T6SS-1, it did not lead to any significant differences in virulence. However, the function of all the factors of T6SS in colonization and antagonism is yet to be explored in detail.

### 1.11 Bacterial structures causing virulence

Structural elements responsible for virulence are crucial for the infection and the pathogenic acclimatization inside the host. Bacterial capsular polysaccharides are important in virulence as they obstruct the actions of host phagocytes [91]. These virulence factors like xanthan, toxins, and adhesion factors are highly accountable for the pathogenicity of *Xanthomonas*.

**EPS Xanthan:** The pathogenic factors in *Xanthomonas* include exopolysaccharides (EPS) that form biofilms. Xanthan, EPS, is a distinctive feature found in all Xanthomonads, giving rise to the mucous or mucoid appearance of the bacterial colonies which is a major component of its biofilm matrix [92]. Xanthan is made of cellulose-like (1 → 4)-β-d-glucose, containing a (1 → 3)-linked trisaccharide side chain of β-*d*-mannose-(1 → 4)-βD-glucuronic acid-(1 → 2)-α-d-mannose. Xanthan's capacity to shield bacteria from environmental challenges like desiccation and harmful substances stems from its high-water content and anionic nature. However, xanthan, in vascular pathogens, can lead to blocking of water flow in xylem vessels resulting in the wilting of host plants [93,94]. It also suppresses callose deposition inducing plant susceptibility to *Xanthomonas* [95]. Biofilms formed by *Xanthomonas* secure it from host defense response and antibiotics produced. Furthermore, it likely controls the bacteria's survival on the plant's surface before entering the intercellular space, or it may affect the adherence of vascular bacteria to xylem vessels [96]. Several genetic loci such as the highly conserved *gum* gene cluster (consisting of a total of 12 genes from *gumB* to *gumM*) direct the production of xanthan [97,98].

**Toxins:** Among *Xanthomonas* spp., the production of the toxin is presumed to be constrained to the *X. albilineans*. *X. albilineans*, the causal organism of leaf scald disease in sugarcane, lacks T3SS and also does not produce xanthan gum [99]. However, it secretes phytotoxin albicidin, which is a DNA gyrase inhibitor and is synthesized by nonribosomal peptide synthases [100]. It is synthesized by NRPS-PKS (Non-Ribosomal Peptide Synthetase-Polyketide Synthase). Albicidin inhibits chloroplast formation, resulting in the whitening of leaves. Nevertheless, it was not the predominant factor affecting the prevalence of *X. albilineans* since mutants lacking albicidin retained their virulence [101]. Moreover, some *Xanthomonas* spp. like *X. oryzae* pv. *oryzicola* and *X. axonopodis* pv. *citri* encode genes for the NRPS enzymes. These are much similar to phytotoxin syringomycin, which is encoded by syringomycin synthetase genes [28]. There is ample opportunity for comprehensive research to clarify the correlation between the presence of NRPS coding genes and toxin secretion in *Xanthomonas* species, as it remains unclear at present.

***Xanthomonas* LPS:** LPS (lipopolysaccharides) are tripartite glycoconjugates in which the glycolipid part is known as lipid A. It carries a core oligosaccharide and a polysaccharide (O-antigen), which constitutes the outer layer of the outer membrane. Additionally, rough-type LPS or lipooligosaccharides (LOS) are LPS lacking O-antigen components. LPS serves as a protective barrier against antimicrobial substances that enhance the pathogen growth. It is also critical for the outer membrane's structural properties. LPSs also mediate adhesion and host recognition [102]. Defense-related responses are observed to be induced by LPS belonging to different *Xanthomonas* spp. in plants [103,104]. The ability of lipid A in *X. campestris* to stimulate the innate immunity in the host plant (*Arabidopsis thaliana*) is governed by a pattern of acylation and phosphorylation [105]. Lipid A is conserved throughout the Gram-negative bacteria. Among core oligosaccharides, more variation is observed. Also, structures of the O-antigen of *Xanthomonas* species are specific. However, to date, the structural details of only a few LPS have been determined.

**Adhesins:** Adhesion to the biotic surface is the initial step toward bacterial invasion in the host plant tissues. The adhesins anchored in the bacterial outer membrane influence bacterial attachment. These adhesins are classified into fimbrial and nonfimbrial adhesins. Non-fimbrial adhesins consist of XadA and XadB (homolog to adhesin YadA and the autotransporter YapH from *Yersinia* spp.), FhaB (filamentous hemagglutinin-like proteins). These adhesins help in *Xanthomonas* attachment on leaves as well as entry inside the host cell. Fimbrial adhesins consist of type IV pilus secretin PilQ. Gerlach and Hensel [29] reported that the predicted structure of the periplasmic pilus of T2SS systems matches the filamentous proteinaceous structure of type IV pili [106,107]. These aids in *Xanthomonas* replication and spread of infection in host tissue. Up to now, adhesins in *X. oryzae* pv. *oryzae*, *X. fuscans* ssp. *fuscans* and *X. axonopodis* pv. *citri*, and have been demonstrated to play a role in bacterial virulence and in the attachment to biotic surfaces [[108], [109], [110]]. It is well known that bacterial adhesin binds to host surface receptors, but the virulence function is intensively studied in animal pathogenic bacteria, but it is lesser known in plant pathogenic bacteria.

### 1.12 Ice-nucleation activity

Discovery of ice-nucleation activity in bacteria was done in phytopathogen *Pseudomonas syrigae* during the 1970s. Following this, researchers identified several other ice-nucleating bacteria belonging to families Pseudomonadaceae, Enterobacteriaceae, Xanthomonadaceae, and Lysinibacillus [111]. *Xanthomonas* spp. too possess ice-nucleation activity, enabling them to initiate ice formation even at relatively high temperatures. Freezing injury tends to increase the likelihood or severity of disease and the freezing/thawing process aids in the passive entry of pathogens as reported for *Pseudomonas syringae* into cherry leaves [112]. Azad and Schaad [113] found that wheat, barley, beans and corn plants inoculated with *X. campestris pv. translucens* exhibit increased susceptibility to frost damage compared to control plants irrigated solely with water. Frost-exposed plants (−3 °C) showed faster lesion development than those unexposed. Moreover, the number and size of lesions increased with longer incubation periods between inoculation and freezing. *X. campestris pv. translucens* strains isolated from diseased plants exhibited ice nucleation activity within the temperature range of −2 to −8 °C. This ability of bacteria to induce ice formation is attributed to the specialized proteins that are anchored to the outer membrane of the bacterial cell [114]. As a plant pathogen, *Xanthomonas* spp. exacerbate frost injury to plant tissues by elevating the nucleation temperature of water, thereby gaining access to nutrients within the plant. The ice nucleation activity of *X. campestris pv. translucens* shows both quantitative and qualitative variations. A significant correlation was observed between the level of ice nucleation activity and the extent of frost injury in inoculated maize seedlings at −5 °C [114].

### 1.13 Control mechanisms

*Xanthomonas* causes complex diseases and needs intense strategic management. Most of the crop plants are susceptible towards phytopathogens. Disease control mechanisms can include cultural practices, chemical control, biological control, and host genetic resistance. The development of disease-free seeds, resistant varieties, strict phyto-sanitation, and bactericidal applications can play key management against *Xanthomonas* infection. Chemical, biological, and biotechnological tools can be of substantial use in the control mechanism which are discussed in the following sections.

### 1.14 Chemical control

Professor Millardet in 1882, developed Bordeaux mixture that constituted of copper, lime, and sulfate significantly controlled grape downy mildew [115]. The recommended dose of metallic copper was 0.5–0.7 g/L under field conditions [116]. Gradually copper became a key component in pesticides for plant disease control, which effectively induced and killed persistent cells of *Xanthomonas* [117]. Later research by Martins et al. [118] unveiled that proline functions as a signaling molecule that triggers the reawakening of *X. citri*. However, contrary to this, isoleucine had an inhibitory impact, obstructing the revival of persisters even when proline was present. Along with extensive copper use, in the 1950's the dominant bactericide used was streptomycin to treat *Xanthomonas* infection, but gradually streptomycin-resistant *Xanthomonas* strains developed [119]. Further prolonged use of copper resulted in copper-tolerant *Xanthomonas* strains in the late 1960s [120]. The addition of ethylene-bis-dithiocarbamates along with copper bactericides came into practice to check bacterial spots caused by *Xanthomonas* [121]. It was also reported that compared to basic copper sulfate, applying a mixture of copper and mancozeb effectively controlled *Xanthomonas* [121]. Mancozeb increases copper solubility hence increasing its effectiveness in infection control. Presently, commercial copper bactericides constitute micron-size metallic insoluble copper in the form of copper (II) hydroxide, copper (II) oxide, and copper (II) oxychloride [122]. Kocide 3000 is a commercially available bactericide marketed by DuPont, Wilmington, DE, which contains micron-size metallic copper (II) hydroxide (5 μM) [119].

Further control of *Xanthomonas* biofilm formation can be effectively prevented by the treatment of d-leucine and 3-indolyl acetonitrile (IAN) [123]. Carvacrol, extracted from essential oils of *Zataria multiflora*, is reported to effectively control phytopathogens It leads to a notable reduction in the intensity of bacterial leaf spot disease in tomatoes and enhances the effectiveness of copper treatment against copper-resistant strains of *X. euvesicatoria* pv. *perforans* [124]. Acibenzolar-S-methyl (ASM) acts as a plant activator that induces ISR in pepper and tomato. Weekly foliar treatment showed alleviation in bacterial leaf spot in pepper [125]. Another substitute to copper treatment is the use of zinc as it also has antimicrobial properties and is a safer product. A zinc-based formulation, Zinkicide, has been reported to reduce 38–42 % of the canker lesions in pineapple leaves under greenhouse conditions [126]. The use of zinc lowers the metal accumulation in soil, unlike copper. Ternary solution (TSOL) constituted of zinc, salicylic acid, and hydrogen peroxide, is reported to be effective against *X. citri* [32]. Presently, a hybrid nanoparticle developed from copper-zinc is reported to exhibit antimicrobial activities against phytopathogens. Foliar treatment with benzoic acid has also led to an increase in resistance against *X. campestris* pv. *campestris*, which causes black rot disease in cauliflower [127]. *X. campestris* pv. *campestris* inoculated *Brassica rapa* when sprayed with 3,4-(methylenedioxy) cinnamic acid (MDCA), effectively enhanced disease emergence [128] as MDCA acts as the inhibitor of 4-coumarate–CoA ligase which is responsible for the formation of hydroxycinnamic acids and flavonoids.

Although chemical controls provide quick prevention against plant diseases, in the long run, it results in bio-accumulation, soil nutrient loss, pathogenic resistance and many more undesirable effects. Biological control as an alternative promising disease management strategy as it is environmentally sustainable and economically viable.

### 1.15 Biological control

**Fig. 3:**
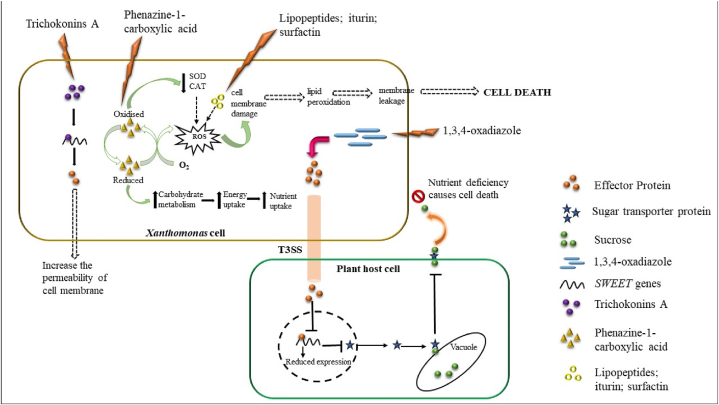
Biocontrol mechanisms of *Xanthomonas* infection.

Biological control is one of the eco-friendly approaches under the integrated disease management techniques. Microorganisms residing in plant rhizospheres and phyllosphere have a great role in disease management. They secrete antimicrobial or SAR-inducing compounds that help to induce host plant immunity. Plant-associated microorganisms are attractive biocontrol agents and need to be explored critically. Before implementing biocontrol, especially in situations of severe disease spread, it is crucial to gain a deeper understanding of the relationship between plants, pathogens, and environmental factors. In addition to the microbial applications, the plant extracts can be utilized in the form of biofertilizers and biopesticides as well as gene products also serve as effective biological control agents (BCA). Bioactive compounds derived from bacteria and fungi play a significant role in regulating the growth and infection of *Xanthomonas* in the host plant. They achieve this in pathogen by generating reactive oxygen species, causing DNA damage, enhancing cell permeability, or by interfering with the nutrients transport to *Xanthomonas* from host as depicted in Fig. 3.

### 1.16 Bacterial control

Bacteria inhabiting the rhizospheric surface of plants are proficient in repressing phytopathogens as well as growth promotion of the host plants. These beneficial bacteria contribute to the natural defense mechanism of host plants from phytopathogens by synthesizing organic compounds having antimicrobial activity (direct mechanism) or by inducing and improving plant immunity (indirect mechanism). A lot of work has been done to identify such rhizospheric and endophytic bacteria and exploit them in combating *Xanthomonas* infections in the host plants. These bacteria enhance competition, parasitism, and antibiosis, as well as produce bioactive compounds that stimulate SAR (Systemic Acquired Resistance) and ISR (Induced Systemic Resistance). Pathogen cell lysis is mediated by hydrolytic enzymes like cellulase, chitinase, glucanase, and protease produced by the beneficial bacteria. Antibiosis occurs through the production of various substances such as antibiotics like oomycin A, 2,4-diacetyl phloroglucinol (DAPG), pyoluteorin, phenazine-1-carboxamide, phenazine-1-carboxylic acid, pyrrolnitrin, as well as organic compounds, HCN, toxins, and hydrolytic enzymes like β-xylosidase, chitinase, pectin methylesterase, and β-1,3-glucanase. These substances break down glycosidic linkages in the bacterial cell wall, aiding the host plant in its defense against pathogens [129,130]. Some of the bacterial bioactive compounds responsible for the inhibition of *Xanthomonas* are listed in Table 1.

**Table 1:** Bioactive compounds isolated from bacterial extracts showing inhibitory activity against *Xanthomonas* spp.

S. No.	Bacteria	Bioactive Compound	Pathogen	Disease	References
1.	*Pseudomonas aeruginosa* VRKK1	Octadecanoic acid 2-oxo methyl ester	*X. campestris*	Bacterial blight in cowpea	[131]
2.	*Bacillus velezensis* ELS2	E−4-Bromo-3-methyl-1-(tetrahydro-2-pyranyloxy)-2-butene	*X. citri* pv*. malvacearum*	Bacterial blight in cotton	[132]
3.	*Bacillus amyloliquefaciens* FZB42	Difficidin; Bacilysin	*X. oryzae* pv. *oryzae*	Bacterial blight in rice	[133]
4.	*Bacillus velezensis* 25	Bacillomycin-D	*X. citri* subsp. *citri*	Citrus canker	[134]
5.	*Bacillus albus* CWTS 10	2-deoxystreptamine; Miserotoxin; Fumitremorgin C; Pipercide; Pipernonaline; Gingerone A; Deoxyvasicinone	*X. oryzae*	Bacterial leaf streak in rice	[135]
6.	*Pseudomonas fluorescens*	2,4-diacetylphloroglucinol	*X. campestris* pv *campestris*	Black rot disease in cabbage	[136]
7.	*Bacillus velezensis* HN-2	C_15_ surfactin A	*X. oryzae* pv. oryzae	Bacterial rice leaf blight	[137]
8.	*Pseudomonas entomophila*	Diketopiperazines	*X. citri* subsp. *citri*	Citrus canker	[138]
9.	*Pseudomonas mosselii* strain 923	Pseudoiodinine	*X. oryzae* pv. *oryzicola*	Bacterial leaf streak	[139]
10.	Consortia of *Priestia megaterium* T3 and *Bacillus cereus* T4	2-thio-5(N-methylaminomethyl) uridine, Diprotin A (Isoleucyl Prolyl Isoleucine), Plipastatin, Gramicidin, Kurstakins, Fusaricidins, Surfactin, Polymyxin	*X. vesicatoria*	Leaf spot in tomato	[140]
11.	*Paenibacillus peoriae* strain To99	Polymyxin A	*X. perforans T4*	Bacterial spot of tomato	[141]
12.	*Pseudomonas aeruginosa* strain BRp3	1-hydroxy-phenazine, pyocyanin, pyochellin, rhamnolipids, 4-hydroxy-2-alkylquinolines, 2,3,4-trihydroxy-2-alkylquinolines and 1,2,3,4-tetrahydroxy-2-alkylquinolines	*X. oryzae* pv. *oryzae*	Bacterial Leaf Blight in Basmati rice	[142]
13.	*Pseudomonas orientalis* X2-1P	phenazine-1-carboxylic acid	*X. campestris* pv. *campestris*	Black rot in oilseed rape	[143]
14.	*Streptomyces* sp. AN090126	2-propyl furan, 3-methyl-1-butanol, 2-methyl-1-butanol, and dimethyl disulfide	*X. euvesicatoria*	Leaf spot in red pepper	[144]
15.	*Paenibacillus yonginensis DCY84T*	hydroperoxy-dihydroxy-octadecenoic acid, tricin hexoside, corchorifatty acid F, pinellic acid, dihydroxy-octadecadienoic acid and dihydroxy-octadecenoic acid	*X. oryzae* pv. *oryzae*	Bacterial blight in rice	[145]

*Bacillus* spp. is widely known for its biocontrol activity [146]. The treatment of *B. subtilis* TKS1-1 and *B. amyloliquefaciens* WG6-14 resulted in reduced colonization and diminished biofilm formation by *X. citri* subsp. *citri* which in turn, led to a decrease in the development of citrus canker disease [147]. The production of lipopeptides including iturin, bacilysin, surfactin, bacillomycin, zwittermicin, and fengycin has been reported in *Bacillus* spp. which plays an active role in antibiosis [148]. It is well documented that foliar treatment of naturally isolated strains of *Bacilli* and *Streptomyces* significantly decreases *Xanthomonas* infections and symptom reduction in various crops when tested in field conditions [149,150]. Pajcin et al. [151] reported that several BCA, including *Lactobacillus* MK3, QM 9414, and *P*. *aeruginosa* 1128, exhibit substantial antibiosis capabilities against *X. euvesicatoria* pv. *euvesicatoria*, which is responsible for bacterial leaf spots in chili and tomato plants. Up-to 65 % reduction in symptoms was observed in inoculated *B. velezensis* IP22 pepper leaves in *X. euvesicatoria* pv*. euvesicatoria* infected plants. This was due to the lipopeptides from fengycin and locillomycin families produced by *B*. *velezensis* IP22 [151]. Mishra and Arora [136] have evaluated fifty-four rhizo-bacterial strains of *B. campestris* for their antagonism against *X. campestris pv. campestris*. Among them, two isolates, *P. aeruginosa* KA19 and *B. thuringiensis* SE, exhibited inhibition halos wider than 11 mm. Both isolates applied separately and in combinations with various techniques (foliar spray/seed soak/soil drench) performed well in controlling black rot disease. When used as a mixture, the results were significantly better. Seed priming with rhizobacterial strains can stimulate phenylpropanoid and isoflavonoid pathways by accumulating isoflavonoid phytoalexins and phenolic compounds. This leads to the improvement of the biocontrol mechanism as well as promotes plant growth [152]. A significant reduction of about 50 % is observed in bacterial leaf spots severity when humic acids along with *H. seropedicae* suspension (10^8^ cfu/ml) was used in tomato [153]. Le et al. [154] reported that treating chili with *Paenibacillus elgii* significantly decreased the bacterial leaf spot by 67 %. Tomato plants when treated with *hrpG* mutant strain of *X. euvesicatoria* pv. *euvesicatoria* 75-3S showed a decrease of 57 % compared to control plants for leaf spot disease under both greenhouse and field conditions [155]. Some of the bacteria like *B. subtilis* strain QST 713 are commercially available under the trademark of Serenade® OPTI (BAYER) which are used against *Xanthomonas* infection. The *Bacillus subtilis* group also secretes nitrogen-containing VOCs that can be categorized based on their cyclization rate. Among the cyclic compounds, azoles are the predominant group exhibiting antimicrobial properties [156]. Azole groups are also used in the agronomy sector to design effective fertilizers. 1,3,4-oxadiazole is a potential oxadiazole-based agrochemical that induces the starvation mechanisms in *Xanthomonas* spp. by manipulating the type III secretion system. It may indirectly suppress the inducible activation of host *SWEET* genes (Fig. 3), thus mitigating sucrose availability during the infection process to the phytopathogenic bacteria, thereby being used as a biocontrol agent in agriculture [157]. Phenazine-1-carboxylic acid which is a bioactive compound of *Pseudomonas* is formulated under the trade name of shenqinmycin and is globally used as a biopesticide to control *Xanthomonas* infection. *Bacillus subtilis* strain QST 713 (1.34 %) is formulated under the trade name Cease®, which is used to treat bacterial spot caused by *Xanthomonas* spp and gray mold caused by *Botrytis cinerea*. *Bacillus subtilis* strain IAB/BS03 (0.08 %) is formulated under the trade name AVIV®, which is used to treat bacterial spot caused by *Xanthomonas* spp. These are used commercially as a crop protectant worldwide.

### 1.17 Fungal control

The plant-fungus symbiosis exhibiting great diversity and ubiquity is an important interaction in ecological functioning. Studies have shown that some symbiotic fungi which are crucial in plants adapting towards stress can be exploited as fungal bio-control agents [158]. These may include *Trichoderma* spp., arbuscular mycorrhizas (AMF), yeasts, ectomycorrhizas, and fungal endophytes. They show significant antagonism against fungal pathogens including *Alternaria*, *Candida*, *Aspergillus*, *Fusarium*, *Pichia*, *Penicillium*, *Pythium*, *Verticillium* and *Talaromyces*, which cause widespread diseases in host plants. Several mechanisms are applied by these fungal biocontrol agents against different pathogens. *T*. *harzianum* exhibits parasitism against *S*. *sclerotiorum* by degrading the hyphal growth of the pathogens [159]. Likewise, *P*. *lilacinum* performs antibiosis, by secreting various extracellular enzymes to inhibit white mold [160]. *P*. *lilacinum* secrete extracellular enzymes which digests the eggs and parasitize the egg-laying female nematode, *Meloidogyne enterolobii* [161]. *Trichoderma* spp. are filamentous fungi found in the soil, renowned for their effectiveness in a wide range of applications that promote plant health and benefits [162]. These strains utilize a sophisticated mechanism for controlling pathogens like mycoparasitism [163,164], competing for physical space for root colonization, preventing the proliferation of phytopathogens by cell lytic enzymes secretion, antagonistic secondary compounds production, plant defense responses stimulation, enhancing plant growth, and increasing plant resilience against both biotic and abiotic stress factors [165].

*T. harzianum* and *T. strigosum* are reported to protect tomato against *X. euvesicatoria* causing leaf spot disease effectively [166]. *Trichoderma* produces terpenoid phytoalexins that is responsible for the antagonistic behavior towards the phytopathogens [167]. *Trichoderma longibrachiatum*, is efficient against *X. campestris* pv. *campestris* and *X. arboricola*, by secreting trichogin GA IV peptaibol, which possesses antimicrobial activity [168]. Zhang et al. [169] reported in their study the antibacterial effects of Trichokonins A and peptaibols produced by *Trichoderma longibrachiatum* SMF2 to control the infection of *Xanthomonas oryzae* pv. *oryzae* causing bacterial leaf blight on rice (Fig. 3). Consortia of *T*. *asperellum* strain ICC 012 and *T*. *gamsii* strain ICC 080 (2 % + 0.08 %) formulated under the trade name Bio-Tam®, is used to treat wilt caused by *Xanthomonas* spp. This is also used commercially against *Phytophthora, Rhizoctonia, Pythium* and *Verticillium*.

Besides *Trichoderma* spp., *Arbuscular* mycorrhizal fungi (AMF) are the most prevalent, holding the largest biomass and having an antagonistic effect on phytopathogens, making it a promising beneficial fungus [170]. A notable increase in defense-related transcription factors in *Medicago truncatula* when infected with *X. campestris* indicates that AMF symbiosis is responsible for the induction of ISR response [171]. In *X. campestris* pv*. oryzae* infected rice plants causing bacterial blight it is observed that AMF colonization at the seedling stage is lowest and enhances significantly with maturity imparting a high degree of resistance towards the pathogen during maturity re-establishing the fact that percent AMF colonization plays a direct role in biotic stress management [172].

Yeast is also an effective biocontrol agent thriving with minimal culture requirements and posing limited biosafety concerns. *A. pullulans*, *C. albidus*, *C. oleophila*, *S. cerevisiae*, and *M. fructicola* are broadly used as BCA. The mechanisms used by them to combat pathogens are competition, secretion of volatiles, mycoparasitism, enzymes and toxins production, and stimulation of phytoimmunity in host. Black rot of crucifers caused by *X. campestris* pv. *campestris* have been reported to be biologically controlled using epiphytic yeast isolated from cabbage leaves with 72–79 % reduction in disease severity [173]. It is also reported that edible strains of *S. cerevisiae* NJ-1 isolated from the pomace of *Ficus carica* secrete 3-methyl-1-butanol, a volatile organic compound that inhibits the production of aflatoxin [174]. Yeast can be used as an active biocontrol agent but the paucity of exploration and exploitation limits its full utilization.

### 1.18 Phage-based biocontrol

An integrative disease management system has picked up interest in applications of phage-mediated *Xanthomonas* biocontrol in agricultural fields. Bacteriophages are viruses that infect and replicate within bacterial cells. They are recognized as the most abundant biological entities on the planet and are extensively distributed in the environment. The replication cycle consists of temperate and lytic phases. The lytic pathway is a pivotal element in biocontrol activity. In this pathway, bacteriophages adhere to the bacterial surface, introduce their genetic material, and replicate within the pathogen cell. This ultimately leads to the multiplication of phage, which cause lysis and the demise of the pathogen [175]. In the 19th century, the biocontrol activity of phage drew attention of researchers, when a filtrate derived from decomposing cabbage was used, it effectively halted the spread of cabbage-rot disease induced by *X. campestris* pv. *campestris* [176]. Later, Civerolo and Kiel [177] reported a similar experiment to check bacterial spot caused by *X. campestris* pv. *pruni* in peach. It is now widely progressed in field trials as well as explicit research is carried out on phage biocontrol mechanisms. Bacteriophage alter the mechanism of DNA methylation, phosphorylation, and transcription in the host cell. In the case of Phage Xp12 infecting *X. oryzae* pv. *oryzae*, this infection prompts the production of 5-methylcytosine, replacing all cytosine residues only in the DNA of Xp12 resulting in change of the physical and chemical properties of DNA, like low buoyant density and high melting temperature ultimately causing death of the cell. Phage biocontrol is widely used against bacterial spot of tomato caused by *X. campestris* pv. *vesicatoria* [178], geranium bacterial blight caused by *X. campestris* pv. *pelargonii* [179], *X. axonopodis* pv. *allii* causing leaf blight of onion [180], *X. axonopodis* pv. *citri* and *X. axonopodis* pv*. citrumelo* causing citrus canker and citrus bacterial spot [181], *X. axonopodis* pv. *citri* causing Asiatic citrus canker [182], *X. oryzae* pv. *oryzae,* causal organism for bacterial leaf blight of rice [183] *X. axonopodis* pv. *allii,* causal organism for bacterial leaf blight of Welsh onions [184]. On application of lytic phage cocktails like Xp3-A and Xp3-I on peach seedlings, they resulted in a 51–54 % reduction in symptom emergence of bacterial spot caused by *X. pruni* under environmental conditions [185]. To date, two commercial *Xanthomonas* phage products are available, manufactured by Agriphage™ which are reported to successfully regulate citrus canker disease and tomato and pepper spot disease [186]. Detailed research can result in more commercial products leading to the exploitation of the complete potential of phage as a BCA.

## 2 Conclusion

The efforts invested in understanding *Xanthomonas* ecology, epidemiology, and phytopathology are ultimately aimed at controlling and suppressing plant diseases caused by these bacteria. While there has been notable advancement in comprehending the functions of *Xanthomonas* secretion systems, primarily focusing on T2SS and T3SS substrates, functionally characterizing the protein effectors remains limited in scope. Consequently, ongoing efforts directed at identifying and characterizing effectors produced by the other secretion systems, hold great promise as avenues for unveiling new and unique mechanisms and functions utilized by *Xanthomonas* to manipulate target cells and flourish in their specific environmental niches. This knowledge has started to find practical applications in numerous strategies, including chemical control of pathogens, utilizing biocontrol agents, and developing resistant plant varieties. Furthermore, the increasing recognition of the plant microbiome's role in enhancing plant growth promotion and phyto-immunity raises the possibility of harnessing endophytic and epiphytic microbial communities as potential biocontrol agents, which could be integrated into pest management systems. However, it is important to mention that research on the influence of the microbiome on *Xanthomonas* colonization and strategical disease management is still in its nascent stages and needs to be emphasized minutely.

## Data availability statement

The authors declare that no data associated with our study has been deposited into a publicly available repository since no data was used for the research described in the article.

## Ethics declarations

Review and/or approval by an ethics committee was not needed for this study because it donot include any human or animal participation.

## CRediT authorship contribution statement

**Riddha Dey:** Writing – original draft, Formal analysis. **Richa Raghuwanshi:** Writing – review & editing, Supervision, Resources, Conceptualization.

## Declaration of competing interest

The authors declare the following financial interests/personal relationships which may be considered as potential competing interests:Richa Raghuwanshi reports administrative support and equipment, drugs, or supplies were provided by 10.13039/501100002742Banaras Hindu University. If there are other authors, they declare that they have no known competing financial interests or personal relationships that could have appeared to influence the work reported in this paper.
